# A scoping review and categorization of music and health psychometric inventories

**DOI:** 10.1177/03057356251322071

**Published:** 2025-04-11

**Authors:** Friederike Koehler, Michael J Silverman, Amy Riegelman, Jessica M Abbazio, Suvi Saarikallio

**Affiliations:** 1 Centre of Excellence in Music, Mind, Body and Brain, Department of Music, Art and Culture Studies, University of Jyväskylä, Jyvaskyla, Finland; 2 University of Minnesota, Minneapolis, MN, USA

**Keywords:** music, well-being, assessment, measurement, tool, scale

## Abstract

Healthcare is often dependent on evidence derived from quantitative measurement. Music-based psychometric inventories are thus necessary to quantify health-related constructs. Despite an increase in the number of inventories, there is no systematic overview of the existing inventories, which may hinder dialogue across music disciplines (e.g., music psychology, music therapy). Therefore, the purpose of this scoping review was to identify and categorize psychometric inventories measuring music and health. This pre-registered review followed best practice and was reported following PRISMA guidelines. We extracted data and used a two-phase process to categorize inventories based on our operational definitions. After screening 904 titles and abstracts, we identified 56 psychometric inventories that met our inclusion criteria. Based on full-text reviews, we categorized the inventories into seven groups: Functions of music (16 inventories); Clinical assessment (9 inventories); Music-based intervention (8 inventories); Music engagement (7 inventories); Musicians’ health (7 inventories); Music processing (4 inventories); and Perception of self and others (5 inventories). The inventories captured a wide range of highly specified and diverse approaches to music and health. This overview and categorization may encourage researchers to use the inventories, apply them to a broader range of clinical contexts, and to inform the development of new inventories.

Many people use music to maintain, alter, and promote their health and well-being. As such, music for health promotion has gained growing recognition as a complementary, integrative, or alternative approach to more traditional biomedical treatments such as pharmacology (e.g., Batt-Rawden, 2010). Recent interdisciplinary research examining relationships between music and health has expanded, resulting in various systematic reviews and meta-analyses attempting to collate and quantify the effects of music on aspects of health (e.g., Daykin et al., 2018; Sheppard & Broughton, 2020; Viola et al., 2023). As the interest in the topic of music and health expands, so does the need for related objective measurement. To date, however, no review or overview on the psychometric inventories for assessing music and health exists.

Based on the World Health Organization definition (WHO, 2025), health can be understood as a holistic state that includes optimal physical, mental, and social well-being. This conceptualization of health encompasses more than just the absence of illness and highlights a multidimensional understanding of health on a continuum beyond a dichotomy of health and disease. To provide healthcare policy makers and administrators with the best evidence available and minimize the potential for harm, it is also important to acknowledge the complex and nuanced nature of the relationship between music and health (Musgrave, 2023). Because everyday music engagement can be influenced by individual and contextual factors, people in clinical and non-clinical settings can use music in both adaptive and maladaptive ways (Saarikallio, 2017). Therefore, future research is warranted to investigate the multifaceted aspects of music and health using established psychometric inventories (Koehler et al., 2023).

To increase access to music as a health resource, researchers need to provide evidence to policymakers and administrators. Also highlighting the relevance of evidence in contemporary healthcare, practitioners are often guided by evidence-based practice (EBP). EBP is informed by (1) the best available scientific information; (2) the service user’s preferences and values, and (3) the clinician’s expertise (Sackett, 1998). To address the first aspect of EBP, scholars have created psychometric inventories for use in music research for the quantification of health-related constructs. Such psychometric inventories are valuable in clinical practice and applied research for measuring and evaluating certain health aspects. Psychometric inventories are also vital within the health sciences to advance the understanding of phenomena (Vitoratou & Pickles, 2017). However, some of the existing music and health psychometric inventories were developed and tested solely in non-clinical settings, therefore potentially overlooking their relevance in clinical contexts. In addition, music research is complex as it can be approached from a multitude of disciplines and perspectives including but not limited to music psychology, music education, and music therapy. However, to the best of our knowledge, there is currently a lack of overview regarding psychometric inventories that quantitatively measure music-related health aspects.

To date, there is one review of test instruments in the *Journal of Music Therapy* (Gregory, 2000). However, the absence of a systematic overview of music and health psychometric inventories is consequential because it prevents cross-sectional communication and collaboration as well as comparisons among different contexts and groups. For instance, researchers in music therapy might benefit from employing an inventory developed in music psychology, or music psychologists could build on inventories successfully implemented in music therapy. An overview and categorization of such inventories may therefore offer a valuable opportunity for expanding different perspectives and enriching the quality of research in music and health. It further allows for a more nuanced investigation of relevant psychological processes involved in the relationship between music and health.

The gap in the literature may also limit access to music interventions as non-pharmacological methods for health improvement. Based on the heightened need for psychometric inventories to quantify music and health within contemporary healthcare systems that rely upon quantitative evidence, the aim of the present review is to provide a synopsis and categorization of psychometric inventories measuring music and health. As such, we aim to identify, collate, and categorize existing quantitative inventories since objective measurements are necessary to draw valid conclusions and influence evidence-based research and practice. More generally, our goal is to contribute to an increased recognition of music as a legitimate aspect in healthcare impacting social and cultural policy agendas (Fancourt & Finn, 2019).

## Objective

The purpose of this scoping review was a) to identify the existing music-based health psychometric inventories, b) to categorize the music-based health inventories by type, and c) provide an overview of the scope and content of the inventories.

Our research questions were the following:

1. What psychometric inventories exist that measure music and health?
2. What categories describe the purpose and function of the identified inventories?
3. What constructs do the inventories measure? What subscales do the inventories include? How many items does each inventory have? What population did researchers use to initially test the inventory?

## Method

### Authors’ positionalities and project evolution

Because of the categorization process and operationalization of definitions, we acknowledge the importance of transparency regarding our positionalities. We identify as an interdisciplinary group of privileged White psychologists, music researchers, and librarians from Europe and the United States. After considerable dialogue, we decided to conduct a Critical Interpretive Synthesis (CIS; Dixon-Woods et al., 2006a, 2006b) of psychometric inventories measuring music and health because we anticipated a manageable number of inventories necessary for a CIS. However, based on the number of psychometric inventories that met our inclusion criteria, we pivoted from a CIS to a scoping review as we believed the most appropriate initial step was to identify and categorize the existing psychometric inventories.

### Scoping reviews

Scoping reviews constitute methods for synthesizing large and diverse bodies of literature using systematic and iterative approaches. Although scoping reviews typically do not include an assessment of the quality of the articles meeting inclusion criteria (Grant & Booth, 2009), they can be useful for the preliminary evaluation of the size and scope of a given research topic. By providing a comprehensive overview of broad questions, scoping reviews can incorporate more diverse research methods than systematic reviews of objectivist or interpretivist investigations (Peters et al., 2020). Scoping reviews are typically synthesized using tables and accompanying narrative commentaries (Grant & Booth, 2009). However, because scoping reviews do not evaluate the quality of the evidence, their results should not be used to inform policies (Grant & Booth, 2009). In a scoping review of scoping reviews, Tricco et al. (2016) found that researchers conducted scoping reviews to explore the breadth of the existing literature, map and summarize the literature, and inform future investigations. We thus concluded a scoping review aligned with the existing state of the literature and our goals for this project. Because of our pivot to a scoping review, we decided not to include reliability, validity, strengths, limitations, or comments columns (i.e., assessment of the inventories). Researchers can use scoping reviews to determine if full systematic reviews are necessary (Grant & Booth, 2009). We pre-registered the methods for the present review at the Open Science Framework in April 2023 (https://osf.io/ykzn2/?view_only=92a0fc770f274124b9beb756ccfe1a7d).

### Inclusion and exclusion criteria

We included inventories that were published in peer-reviewed journals in English between January 2000 and May 2023 that measured music related to health. Congruent with the WHO (2025), we conceptualized health as a multifaceted construct that could represent positive/adaptive or negative/maladaptive aspects of health. This included intentional uses of music, functional uses of music, and music as an emotional regulation tool. Inventories had to explicitly use the word “music.” However, no further definitions for the type of music activity were imposed. Therefore, the music activity addressed by an inventory could include any type of musicking, ranging from personal music listening to group playing, therapeutic songwriting, or amateur choir singing. We did not include inventories that used tones or non-music sounds as stimuli. Because of the peer review processes germane to refereed articles, we excluded psychometric inventories that were solely published in books, online or gray literature, or when the inventories were stand-alone inventories including single-item Likert-type scales. We excluded psychometric inventories that were designed to assess emotions conveyed in music as well as music aptitude, music ability, and music preference. We also excluded translations of music and health scales to other languages as well as qualitative interview studies.

### Search methods

In April 2023 a music librarian (J.M.A.) and a social sciences librarian (A.R.), who are both trained on evidence synthesis, designed a comprehensive and reproducible search strategy. The electronic search was informed by term harvesting and testing of known studies featuring music and health psychometric inventories. The librarians selected seven databases with APA PsycINFO (Ovid) as the primary database. The search terms and subject headings targeted the following components: intent, function, or use of music; and psychometric tools. The APA PsycINFO search was then translated to the other relevant databases: Ovid Medline, RILM (Ebsco), CINAHL Ultimate (Ebsco), Music Index Online (Ebsco), Music Periodicals Database (Proquest), and PsyArXiv.

**Figure 1.**
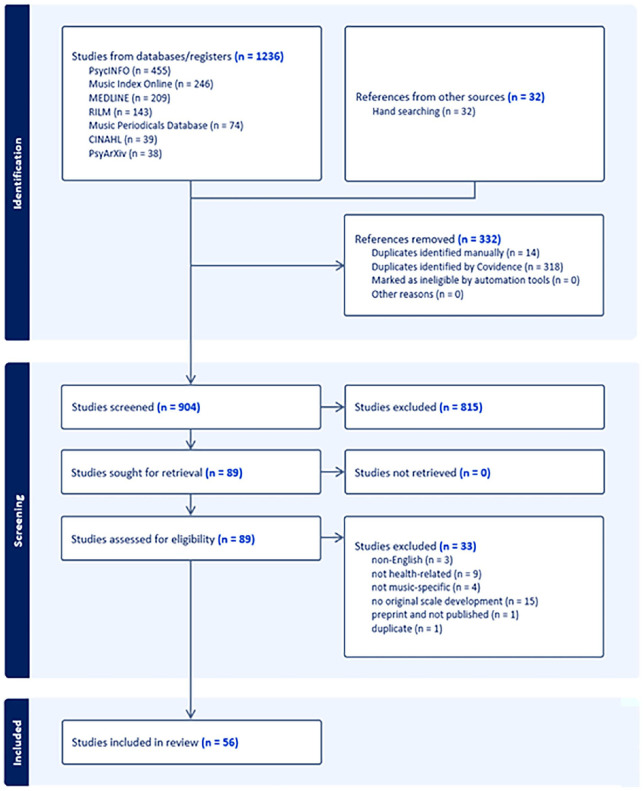
PRISMA Flowchart.

A full search reproducible search strategy is available in Appendix 1. The total number of results from each database is depicted in Figure 1. The librarians then imported the search results into Covidence, an evidence synthesis web application, and deduplicated. Because authors F.K., M.J.S., and S.S., were subject matter experts, they were aware of additional inventories that did not appear in the electronic search strategy results. We therefore used hand searching and also checked the results of our search with Table 1 from Chin et al. (2018) to ensure our search identified inventories that met our inclusion criteria. When we located an inventory that met inclusion criteria, the librarians added those items manually to the Covidence project.

**Table 1.** Functions of Music Inventories.

Scale name	Citation	Construct	Subscales	Items	Population
Adaptive Functions of Music Listening Scale (AFML)	Groarke & Hogan (2018)	Music listening and well-being	Stress regulation; Anxiety regulation; Anger regulation; Loneliness regulation; Rumination; Reminiscence; Strong emotional experiences; Awe and appreciation; cognitive regulation; Identity; Sleep	46	Adults
Barcelona Music Reward Questionnaire (BMRQ)	Mas-Herrero et al. (2013)	Measures how people experience reward from music		20	Professional musicians; non-professional musicians
Brief Music in Mood Regulation Scale (B-MMR)	Saarikallio (2012)	Regulatory strategies in music activities	Entertainment; Revival; Strong sensation; Diversion; Discharge; Mental work; Solace	21	Adolescents
Brunel Music Rating Inventory-2	Karageorghis et al. (2006)	Motivational qualities of music in exercise	NA; Single factor	6	Exercise instructors and participants
Emotion Regulation Strategies for Artistic Creative Activities Scale (ERS-ACA)	Fancourt et al. (2019)	Emotion regular strategies when engaged in artistic creative activities	Avoidance strategies; approach strategies; self-development strategies	18	Adults engaged in creative activities
Eudaimonic Functions of Music Listening Scale	Groarke & Hogan (2020)	Eudaimonic functions of music listening	Transcendence; flow; peak experience	7	Adults
Healthy-Unhealthy Music Scale (HUMS)	Saarikallio et al. (2015)	Healthy and unhealthy music use	Healthy music use; Unhealthy music use	13	Young people
Hedonic and Eudaimonic Motivations for Music Scale (HEMM)	Powell et al. (2023)	Eudaimonic and hedonic motivations for fans of music with violent themes	Eudaimonic and hedonic motivation	12	People who listen to music
Individual and Community Empowerment Inventory (ICE)	Travis & Bowman (2015)	Measures how individuals perceive the empowering and risk enhancing aspects of rap	Individual empowerment; Community empowerment; Individual risk; Community risk	33	High school and undergraduate students
Motives for Listening to Music Questionnaire (MLMQ)	Kuntsche et al. (2016)	Motives for listening to music	Social; Enhancement; Coping; Conformity	16	Adolescents
Music in Mood Regulation (MMR)	Saarikallio (2008)	Regulatory strategies in music activities	Entertainment; Revival; Strong sensation; Diversion; Discharge; Mental work; Solace	40	Adolescents
The Music Use Questionnaire (MUSE)	Chin & Rickard (2012)	Measures an individual’s level of active engagement with music	Cognitive and emotional regulation; Engaged production; Social connection; Dance and physical exercise	58 or 32	Staff and students at a university
Music Mood-Regulation Scale (MMRS)	Hewston et al. (2009)	Mood regulation	Anger; Calmness; Depression; Fatigue; Happiness; Tension; Vigor	21	Sport and exercise population
Musical Activity and Well-being Scale	Krause et al. (2018)	Music participation and health	Mood and coping; Esteem and worth; Socialization; Cognition; Self-actualization	36	People who participated in vocal and instrumental music
Musical Engagement	Hollebeek et al. (2016)	Consumers’ musical engagement	Social identity; Transportive experience, Affect-inducing experience	25	Adults in the United States
Rating of Experienced Social Personal and Cultural Themes of Music Functions (RESPECT-Music)	Boer et al. (2012)	Topography of music functions	Affective and contemplative functions of music; Intrapersonal, social, and sociocultural functions of music	35	Young people in six cultural samples

### Data extraction and category development

After identifying the 56 inventories, we created a spreadsheet for data extraction. Column headers initially included study number, citation, category code for rounds one, two, and three, scale name, construct, subscale/module/domains/factors, number of items, reliability, validity, type of music activity, population (group that the inventory was tested upon), strengths, limitations, and comments. Because of the considerable variance of reliability and validity metrics that authors used and as scoping reviews do not assess the quality of articles (Grant & Booth, 2009), we decided to not include these metrics. Moreover, we only used the original paper describing the inventory for data extraction purposes. We recognize that subsequent analyses, psychometric testing, applications, and translations of these inventories exist, but those were outside the scope of our research questions.

After extracting data from studies meeting inclusion criteria into a spreadsheet, authors FK, MJS, and SS worked collaboratively to determine categories for each of the 56 inventories. This was an iterative, collective, and multi-phased process wherein we repeatedly refined our categorizations to best describe each inventory. Since our aim was to develop those categories based on the included inventories, we followed an inductive data-driven approach to ensure flexibility and a comprehensive use of all data available. In the categorization process, we aimed to find common themes among inventories that were able to differentiate them enough from other clusters. Our goal was to remain as open-minded as possible without having pre-established ideas in mind (e.g., mere building of categories according to music discipline).

We agreed on nine categorizations in this first phase. In the next phase, FK, MJS, and SS developed operational definitions for these categories, merged them when necessary, and then went back through the categorizations of each inventory to ensure each paper was categorized appropriately. In this process, we merged two categories and re-defined our categories. After discussion and refinement, FK and MJS went back through each of the 56 inventories and independently categorized each inventory, resulting in an inter-rater reliability of .89. Authors FK, MJS, and SS then collaboratively resolved all disagreements to arrive at a final inter-rater reliability quotient of 1.00. Throughout the process of categorizing inventories, we did our best to honor the creators’ intentions. We therefore considered the composition of the original paper including but not limited to the purpose of the inventory, subscales/domains, individual items, and the clinical groups and settings.

## Results

We identified 56 psychometric inventories that met our inclusion criteria and somehow addressed music as a health resource. A PRISMA flowchart is depicted in Figure 1.

We categorized the inventories into seven groups based on our operational definitions and categorization processes. Our operational definitions of the seven categories were as follows:

- Functions of music (16 inventories): An inventory that measures how music functions for people (e.g., psychologically, socially, emotionally, physically).
- Clinical assessment (nine inventories): An inventory that is used as an initial clinical assessment to guide further treatment.
- Music-based intervention (eight inventories): An inventory that measures an aspect of a music-based intervention including but not limited to music therapy.
- Music engagement (seven inventories): An inventory that measures how people engage with music.
- Musicians’ health (seven inventories): An inventory that measures aspects of a musician’s health.
- Perception of self and others (five inventories): An inventory that measures aspects of people’s personality or perception of self or others in a music context
- Music processing (four inventories): An inventory that measures how people process music.

The 56 psychometric inventories are listed by categories in Appendix 2. In the following section, we present the main content of each category regarding the scale names, references, constructs that are measured, subscales, number of items, and sample populations.

### Functions of music

We categorized 16 psychometric inventories into the “Functions of music” group, constituting the largest of our seven categories (Table 1). The “Functions of music” group tended to be more heterogeneous than other categories. Some of the inventories aim to capture an overview of a broad range of functions (e.g., RESPECT, MUSE, MLMQ), while most of the inventories have a somewhat more specific focus. The MMR, B-MMR, ERS-ACA, MMRS, and HUMS all focus on music for emotional/mood regulation while the Brunel Music Rating Inventory-2 focuses on exercise and the ICE specifically focuses on rap. Some come from a certain perspective on specific functions (e.g., adaptivity of music: AFML; hedonic/eudaimonic functions: EFML, HEMM; or reward: BMRQ). The number of items ranged from 7 to 58. Some inventories, including the RESPECT and HUMS, were developed with adolescent samples.

### Music engagement

We categorized seven psychometric inventories into the “Music engagement” group (Table 2). This group tended to be homogeneous and often measured music engagement in home environments or with family members. Most inventories focused on music exposure and music engagement in daily activities (e.g., Exposure to Music in Early Childhood Inventory, Music@Home Questionnaire, MusEQ, MEL, MUSEBAQ). The number of items ranged from 14 to 67. Although some inventories were developed with a general population (e.g., Gold-MSI, MEQ, and MUSEBAQ), other inventories were developed for adults with Alzheimer’s Disease or parents of young children on the Autism Spectrum/who identified as Autistic (e.g., MusEQ, MEL).

**Table 2.** Music Engagement Inventories.

Scale name	Citation	Construct	Subscales	Items	Population
Exposure to Music in Childhood Inventory	Cogo-Moreira et al. (2018)	Exposure to music in childhood	Personal music activities; Social music activities	14	Children
Goldsmith’s Musical Sophistication Index (Gold-MSI)	Mullensiefen et al. (2014)	Assessment of musical sophistication	Active engagement; perceptual abilities; musical training; Singing abilities; Emotions; General musical sophistication	38	General population
Music@Home Questionnaire	Politimou et al. (2018)	Home musical environment in early life	Parental beliefs; Child engagement with music; Parent initiation of singing; Parent initiation of music-making	18	Parents and infants
Music Engagement Questionnaire (MusEQ)	Vanstone et al. (2016)	Measure of music engagement in daily life	Daily; Emotion; Perform; Consume; Respond; Prefer	35	Adults with Alzheimer’s disease, neurotypical older adults, informants
Music Experience Questionnaire (MEQ)	Werner et al. (2006)	Individual differences in reactions to music	Commitment to music; Innovative musical aptitude; Social uplift; Affective reactions; Positive psychotropic effects; Reactive musical behavior	53	Community college students
Music in Everyday Life (MEL)	Gottfried et al. (2018)	Parent–child music activities	Joint activities; Routine activities	28	Parents of young children on the autism spectrum
Music Use and Background Questionnaire (MUSEBAQ)	Chin et al. (2018)	Music use and background	musicianship; musical capacity; music preferences; motivations for music use	67	Adults

### Clinical assessment

We categorized nine psychometric inventories into the “Clinical assessment” group (Table 3). This group tended to be heterogeneous because of the varying conditions of different clinical populations. Most inventories focused on a clinical objective that was germane to the symptoms of a given condition including dementia, Autism, and Huntington’s Disease. The number of items ranged from 2 to 88. The inventories were developed specifically for clinical populations, although one inventory was developed with children not in a clinical setting (e.g., MASA-R). These inventories may be especially applicable in certain music therapy and music psychology clinical settings.

**Table 3.** Clinical Assessment Inventories.

Scale name	Citation	Construct	Subscales	Items	Population
Music Attentiveness Screening Assessment, Revised (MASA-R)	Waldon et al. (2016)	Attentiveness to music during a music listening task	Selective and divided attention	2	Children not in a clinical setting
Music-based Autism Diagnostics (MUSAD)	Bergmann et al. (2015)	Autism diagnosis	Social interaction; Stereotypes, restricted, and repetitive behaviors; Motor coordination	original: 88 shortened: 37	Adults with suspected Autism
Music-based Attention Assessment (MAA)	Jeong & Lesiuk (2011)	Music-based attention	Sustained, selective, and divided attention	48	People with traumatic brain injury
Music-based Attention Assessment-Revised (MAA-R)	Jeong (2013)	Music-based attention	Sustained, selective, and divided attention	54	Healthy adults and people with traumatic brain injury
Music Cognitive Test (MCT)	Mangiacotti et al. (2023)	Cognitive abilities stimulated by music making activities	Spatial and time orientation; Language; Memory; Attention; Executive functions, Motor performance	19	Range of people
Music in Dementia Assessment Scales (MiDAS)	McDermott et al. (2015)	Music therapy outcome measure for dementia	Interest; Response; Initiation; Involvement; Enjoyment	7	People with dementia, family carers, home care workers, music therapists
Music Therapy Assessment Tool for Advanced Huntington’s Disease (MATA-HD)	O’Kelly & Bodak (2016)	Music therapy assessment Huntington’s Disease	Arousal/Attention; Physical Presentation; Communication; Musical; Cognition; Psychological/Behavioral	15	People with Huntington’s Disease
Playing-in-Touch Questionnaire (PiT)	Politi et al. (2012)	Musical intouchness in people with “low functioning autism” (p. 552)	NA	10	People with “low functioning autism” (p. 552)

### Music-based intervention

We categorized eight psychometric inventories into the “Music-based intervention” group (Table 4). Similar to the Clinical assessment inventories, this group tended to be diverse because of the various needs of different clinical populations. Most inventories focused on a clinical objective or goal area related to a range of contexts including parent–child dyads, people with acquired brain injury, critically ill patients receiving mechanical ventilatory support, people with dementia or cancer, or hospitalized children. The number of items ranged from 5 to 33. Most inventories were developed with various clinical populations, although one inventory was developed for Guided Imagery and Music Fellows. Similar to the inventories we categorized in the “Clinical assessment” group, these inventories may be applicable in certain music therapy and music psychology clinical settings.

**Table 4.** Music-Based Intervention Inventories.

Scale name	Citation	Construct	Subscales	Items	Population
Assessment of Parenting Competencies, revised (APC-R)	Jacobsen & McKinney (2015)	Parent–child interaction	Mutual attunement; Nonverbal communication; Positive response; negative response; Parent–child interaction in music	5	Parent–child dyads, some with emotionally neglected children
Interpersonal Music-Communication Competence Scale	Hald et al. (2017)	Communicative competencies in music in people with acquired brain injury	Self-disclosure; Empathy; Social relaxation; Assertiveness; Interaction management; Altercentrism; Expressiveness; Supportiveness; Immediacy; Environmental control	30	People with acquired brain injury
Music Assessment Tool (MAT) for Mechanically Ventilated Patients	Chlan & Heiderscheit (2009)	Music preference assessment in critically ill patients	NA; Single factor	13	Critically ill patients receiving mechanical ventilatory support
Music Therapy Engagement Scale for Dementia (MTED)	Tan et al. (2019)	Music therapy engagement	Musical engagement; Relatedness through music; Verbal communication; Emotional response; Extent of overall responsiveness	5 (each item could be a subscale)	People with dementia
Music Therapy Self-Rating Scale (MTSRS)	Meadows et al. (2015)	Measure for cancer patients during music and imagery interventions	Awareness of body; Emotionally focused; Personal resources; Treatment specific	14	Patients with cancer undergoing chemotherapy
The Music Therapy Session Assessment Scale (MT-SAS)	Raglio et al. (2017)	Relationship between service user and music therapist during active music therapy	Items could be considered subscales: Eye contact; Body reciprocity; Countenance and body signals indicating emotional engagement; Refusal/disturbed behavior; Sonorous-musical productions; Attuned sonorous musical productions; Dynamism/variations	7	People with various conditions
Pediatric inpatients music therapy assessment form (PIMTAF)	Douglass (2006)	Pediatric music therapy assessment	Domains germane to pediatrics	33	Hospitalized children
Transpersonal Depth Guided Imagery and Music Inventory (TD-GIM)	Abrams (2016)	Transpersonal depth	NA; Single factor	33 (full version) or 9 (brief version)	GIM Fellows

### Musicians’ health

We categorized seven inventories into the “Musicians’ health” group (Table 5). The inventories cover different components of musicians’ health. Although some psychometric inventories focus on physical aspects (TAPS, EASE, Musculoskeletal Pain Intensity and Interference Questionnaire), others address psychological aspects (MPAI, MARIS). The MOSS and PQRM measured stress related to the occupation. The number of items ranged from 7 to 52 items.

**Table 5.** Musicians’ Health Inventories.

Scale name	Citation	Construct	Subscales	Items	Population
Evaluation of the Ability to Sing Easily (EASE)	Phyland et al. (2013)	Perceived singing voice function	Factor 1; Factor 2	20	Singers
Music Performance Anxiety Inventory for Adolescents (MPAI-A)	Osborne & Kenny (2005)	Music performance anxiety	Somatic and cognitive feature; Performance context; Performance evaluation	15	Skilled young musicians
Musician Occupational Stress Scale (MOSS)	King et al. (2019)	Occupational stress in popular musicians	NA; Single factor	52	Popular musicians
Musician’s arousal regulation imagery scale (MARIS)	Finch et al. (2021)	Arousal imagery strategies in musicians	Mastery; High arousal	20	Professional, student, and amateur musicians
Musculoskeletal Pain Intensity and Interference Questionnaire	Berque et al. (2014)	Pain intensity and interference	Pain intensity; Pain interference	22	Professional orchestral musicians
Psychosocial risks questionnaire for musicians (PRQM)	Jacukowicz & Wezyk (2018)	Psychosocial risks and occupational stress	Job content; Work environment; Work-home interference; Relationships; Lack of stability; Development possibilities; Home-work interference; Tools	31	Musicians
The Technical Ability and Performing Scale (TAPS)	Ramella et al. (2022)	Functional rating scale for musician’s Focal Dystonia	Technical ability; Global performance perception	7	Musicians with Focal Dystonia

### Perception of self and others

We categorized five inventories into the “Perception of self and others” group (Table 6) with considerable heterogeneity among the inventories. Some focus on self-perception related to music in general (MUSPI, MUSCI), while others measure self-perception in specific contexts and samples (caregivers: CCuMS; children and adolescents in a choir: CACES). One addresses perception of others in a musical context (Musical Humility Scale). The number of items ranged from 5 to 36.

**Table 6.** Perception of Self and Others Inventories.

Scale name	Citation	Construct	Subscales	Items	Population
Caregiver Confidence using Music Scale (CCuMS)	Kim et al. (2021)	Caregiver confidence in using music with older adults	NA; Single factor	5	Caregivers of older adults
Children and Adolescent Chorister Engagement Survey (CACES)	Zhukov et al. (2021)	Choir engagement	Self-esteem; Self-efficacy; Identity; Social impact	36	Adolescents in choir
Music Self-perception Inventory (MUSPI; short form)	Morin et al. (2016)	Musical self-concept	Composing; Listening; Dancing; Instrument playing; Reading; Singing	28	Pre-university music students
Musical Humility Scale	Coppola et al. (2021)	Musical humility	Purposeful musical engagement and collaboration; Other-orientedness; Lack of superiority; Acknowledgment of shortcomings and learnability; Healthy pride	30	Undergraduate and graduate students, faculty, music educators
Musical Self-concept Inquiry (MUSCI)	Fiedler & Spychiger (2017)	Musical self-concept in youth	Mood management; Community; Musical ability; Dance and movement; Ideal musical self; Adaptive musical self	28	Secondary education students

### Music processing

We categorized four inventories into the “Music processing” group (Table 7). While some inventories focus on how music is received and perceived emotionally (Music Receptivity Scale, AIMS), others also address aspects related to cognition and appraisal (Music- and Image-related Typicality Scales, Music-empathizing-systemizing). The numbers of items ranged from 3 to 55.

**Table 7.** Music Processing Inventories.

Scale name	Citation	Construct	Subscales	Items	Population
Absorption in Music Scale (AIMS)	Sandstrom & Russo (2011)	Individual’s ability and willingness to let music draw them into emotional experiences	NA; Single factor	34	University students
Music- and Image-related Typicality Scales	Cohrdes & Kopiez (2015)	music- and image-related typicality	Music-related typicality; Image-related typicality	16	Adolescents
Music-empathizing-systemizing	Kreutz et al. (2008)	Cognitive styles of music listening	Music empathizing; Music systemizing	55 (long form); 18 (short form)	Musicians and nonmusicians
Music Receptivity Scale (MRS)	George & Ilavarasu (2021)	Music receptivity	**Long form:** Emotional experience; Interest; Attention; hurdles / **Short form:** Emotion experience; attention	20 **(long form)** or 12 **(short form)**	Adults

## Discussion

Quantitative measurement is a fundamental aspect of clinical practice and evidence-based healthcare (Swan et al., 2023). To advance the music and health discipline, psychometric inventories are necessary to quantify health-related constructs. However, the current lack of a systematic overview of the existing inventories may hinder dialogue and progress across music and health disciplines that ultimately limits access to using music as a non-pharmacological method to improve health. Therefore, the purpose of this scoping review was to identify, synthesize, and categorize psychometric inventories measuring music and health. We identified 56 inventories that met our inclusion criteria. Congruent with the WHO (2025) definition of health as a holistic and multidimensional construct, these inventories reflected a wide conceptual breadth and high research interest in music and health. Our review offers a nuanced overview of the existing inventories and our categorizations recognize the shared qualities of the inventories. This contribution to the literature offers researchers from different disciplines access to and guidance for the application of established psychometric inventories measuring music and health.

Based on our operational definitions and the full-text reviews, we categorized the 56 inventories that met our inclusion criteria into seven groups: Functions of music (16 inventories); Clinical assessment (nine inventories); Music-based intervention (eight inventories); Music engagement (seven inventories); Musicians’ health (seven inventories); Music processing (four inventories); and Perception of self and others (five inventories). Overall, the scope of the inventories covered a wide range of musicking contexts, from music in everyday life to musicians’ health, and the use of music by practitioners in clinical settings. We emphasize the difficulty we experienced developing operational definitions and categorizing the inventories because of the conceptual breadth across as well as the specificity within these inventories. For example, some inventories had multiple subscales that could be categorized into different categories and some inventories could be used in clinical and non-clinical contexts. The various potential applications of the inventories highlight the breadth of the research area and the importance of collaborative dialogue within, as well as the relevance of quantitative measurement in contemporary healthcare policies.

In this scoping review, most inventories meeting our inclusion criteria tended to focus on healthy aspects of music, with the exceptions including the HUMS and the inventories addressing musicians’ health (e.g., MPAI-A, MOSS, Musculoskeletal Pain Intensity and Interference Questionnaire, and PRQM). Similarly, there seems to be a prevailing narrative in contemporary research that music is generally beneficial for health (Musgrave, 2023). However, we contend that the relationship between music and health is more complex than a unidirectional conceptualization. For instance, music can be used as a maladaptive coping strategy (Silverman, 2020, 2021, 2022), can induce substance craving (Short & Dingle, 2016; Silverman et al., 2023), result in rumination (Garrido et al., 2017), depression and worse psychological well-being (Saarikallio et al., 2015), and affective, behavioral, cognitive, identity, interpersonal, physical, and spiritual harm (Silverman et al., 2020). In addition, constructs such as rumination are not necessarily negative and require a more nuanced approach to adequately understand the phenomenon. Perhaps interpretivist paradigms are necessary to better understand the lived experience of certain music and health constructs. Because of the potential for music to have detrimental impacts on health, it is vital that qualified practitioners such as music therapists or other experts in music psychology are involved in decision making when using music in health contexts. Based on the dearth of psychometric inventories that measure maladaptive functions of music, it seems that additional research is warranted to better understand and quantify the deleterious functions of music. We also note that there seems to be an adequate number of existing psychometric inventories in some areas of music measurement (i.e., 16 inventories that measure functions of music). Researchers might dedicate their time and resources to strengthening the literature base by testing inventories in various groups and settings instead of creating new inventories.

With the exception of inventories we categorized into music-based intervention and clinical assessment inventories, many of the inventories were designed for use with and tested in non-clinical populations. To advance the music and health discipline and increase access to treatment for people with various health conditions, researchers will need to use and test psychometric inventories in diverse clinical settings to ensure they are accessible and psychometrically adequate. For example, some self-report outcome measures can be cognitively challenging and may not support recovery (Bibb & McFerran, 2017). Researchers will thus need to be cognizant of the amount and type of language used (Bibb et al., 2016). In addition, inventories used in healthcare contexts and research often measure a negative aspect of health (e.g., pain, depression, anxiety). New inventories developed for clinical populations may therefore balance positively and negatively phrased items to avoid exacerbating negative symptoms. Although conceptualizing health and illness on a continuum may lower stigma (Peter et al., 2021), labeling clinical and non-clinical populations in a binary manner may be detrimental. In addition, populations traditionally termed as non-clinical might also benefit from music research and treatment. For instance, people from communities that have been marginalized for aspects of their identities may not necessarily represent a strictly clinical population yet still merit treatment to augment their health using music.

We also observed a focus on the individual in contrast to the collective in many of the inventories. Although some inventories contained subscales relating to social aspects, only one inventory (CACES) referred to a group music setting. Perhaps resulting from our inclusion criteria regarding the English language, it seems that inventories tended to be designed from an individualized perspective. Based on collectivistic perspectives, researchers might develop inventories that specifically measure music and health in group or social contexts. Furthermore, most of the inventories were designed without a theory-driven approach to health, which might have led to an under- or over-emphasis of certain components of health. For instance, although many inventories addressed emotional health, we did not identify spiritual health as a noteworthy aspect in other conceptualizations (Els & De La Rey, 2006; Myers et al., 2000). In addition, the inventories we identified tended to focus on specific musical settings and activity types such as music listening and clinical music use. However, other contexts, including but not limited to music education and community music (e.g., MacGlone et al., 2020), may be relevant for music and health research and might benefit from the use of such psychometric inventories.

With increasing interest in non-pharmacological and accessible treatments such as music in healthcare settings, the inventories we identified could be relevant for studies aiming to influence healthcare policies and access to treatment (Baker & Young, 2016). Because systematic reviews and meta-analyses incorporating psychometric inventories often drive decision- and policy-making in healthcare (Guyatt et al., 2011), our scoping review may encourage trials using music-related psychometric inventories. However, EBP, healthcare, and policy-making tend to privilege objectivist research (Aigen, 2015). Congruent with rationales for participatory research (e.g., Vaughn & Jacquez, 2020), we argue that individuals’ values, identities, and preferences are vital and need to be considered when developing inventories, designing interventions, conducting research, and making policies. Including people’s values, identities, and preferences is also congruent with EBP (Sackett, 1998). Moreover, for the music and health discipline to continue its establishment in healthcare, economic analyses including cost-effectiveness analyses, cost-benefit analyses, and cost-utility analyses incorporating psychometric inventories are necessary (Else, 2016).

### Limitations

Although we adhered to rigorous and transparent processes in this scoping review and categorization, there are several limitations to consider. First, scoping reviews are designed to address the breadth of a given topic and therefore may lack depth (Tricco et al., 2016). Second, despite our aim to be as inclusive as possible, we recognize that limiting our inclusion criteria to refereed journals in English privileges certain scholars and ensuing conceptualizations of music and health. As such, there is a need for outcome measures to be translated to other languages (Ridder et al., 2017). In addition, the search algorithms and databases we used may have overlooked other relevant publications. Although we used term harvesting and benchmarking to design a comprehensive search, there could have been inventories that we did not identify. This could have resulted from irregular indexing and titles and abstracts lacking key details. We therefore used hand searching in addition to electronic search strategies. Moreover, we only used the original paper introducing the inventory and acknowledge that other researchers may have tested psychometric properties of the inventories or used the inventories in translational research within clinical settings. Furthermore, we did not include risk of bias or other assessments of the included inventories. Although we initially considered reporting reliability and validity, the considerable variance of the metrics authors reported made it unfeasible. Therefore, researchers interested in using this review as a resource to identify specific inventories are advised to make further inquiries into the psychometric properties. In addition, since health can be understood as a multidimensional construct (WHO, 2025), the conceptualization of an inventory into a single category was challenging and prone to subjective bias because of subscales, differing ways of approaching health, and our positionalities.

### Future research and implications

Given the importance of measurement and interest in music as a non-pharmacological treatment (Edwards et al., 2023), there are many areas for future investigation. For example, it might be interesting to identify translations of the inventories as well as to translate the existing inventories into languages that have been marginalized. To remedy a notable limitation of the current study, future researchers might consider studying all the psychometric properties of a single inventory. Furthermore, reviews and meta-analyses of all studies using a specific inventory might provide a valuable contribution to the field. As service users’ values and preferences constitute a component of EBP, it would be interesting to interview various constituents about certain inventories to determine their application for people with various health conditions. Furthermore, translational research should center participants’ experience, perceptions, and recommendations. To advance the literature, researchers creating new psychometric inventories should adhere to Consensus-Based Standards for the Selection of Health Measurement Instruments (COSMIN; www.cosmin.nl) guidelines (Waldon, 2016). In future inventories regarding music and health, researchers will need to address aspects of people’s identities including but not limited to gender expression, ethnicity, religion, and membership in groups that have been oppressed and marginalized to promote representation and belonging. In addition, inventories that measure detrimental aspects of music are warranted as music can impact health in numerous ways. Future reviews might also analyze inventories by the type of music activity, such as listening or active music making.

Our findings offer new directions for future studies and also provide researchers with an overview of the existing tools to optimize their choice of measurement for a specific research question or population. Furthermore, investigators might benefit from selecting and using a suitable measurement developed in other music disciplines to enhance their own field. For instance, a music therapy scholar may notice that a specific music processing scale as used in music psychology might be useful to integrate into their research. In addition, based on this review, relevant measurement gaps in music and health research might be identified and further developed. For example, a researcher with an interest in music engagement and depression may find that the existing inventories do not depict a specific facet of depression and therefore potentially pursue the development of a new inventory.

This scoping review also has implications for practitioners using music to maintain and augment health. For instance, music therapists could use this overview of measurement inventories to discern their usability in their own practice. Inventories developed in other fields might hold great potential to enrich their work. For example, practitioners could use inventories assessing everyday use of music to better understand how people use music throughout their day to modify moods and promote health. Furthermore, as the intentional and functional applications of music within a therapeutic relationship are some of the features that distinguish music therapy from other helping professions, some of the inventories can be used to highlight the unique and comprehensive academic and clinical training of qualified music therapists (Sena Moore, 2015). Researchers might also use the identified inventories to differentiate music therapy from other allied health professions. Although music therapy researchers typically measure non-music health outcomes that may be valued by the medical community (Gregory, 2000), using music inventories as dependent measures in comparative studies might identify the unique components that make music therapy distinct and effective. Music psychologists might use the identified inventories for basic research that music therapists can then translate into clinical settings.

## Conclusion

We identified many music and health psychometric inventories in this scoping review, yet the inventories were highly specified and diverse. This overview and categorization of inventories might encourage music and health researchers to use the inventories, apply them to a broader range of clinical contexts, and to inform the development of new inventories. The development of music inventories with strong psychometric properties has the potential to advance the music and health discipline and ultimately increase access to music as a health resource.

## Appendix 1

### Database search strategies

PsycINFO (Ovid)

1. (music*).ti, ab,id,jx
2. exp Music/ OR Musicians/ OR exp “Music Perception”/
3. (“use*” OR usage* OR function* OR intention* OR intent? OR utiliz*).ti, ab,id
4. exp Intention/
5. (Psychometric* OR (psycholog* ADJ3 (metric* or test* or measure* or tool* OR instrument* OR scale* OR questionnaire*))).ti, ab,id
6. exp Psychometrics/
7. (1 OR 2) AND (3 OR 4) AND (5 OR 6)
8. limit 7 to yr =”2000-2023”
9. 8 NOT (“Dissertation Abstract” or “Book” or “Edited Book” or “Authored Book”).pt.
10. 9 NOT (Dutch or Spanish or French or “Serbo-Croatian” or German or Portuguese or Japanese or Italian).lg

*n* = 455

20230405

Ovid Medline All

1. music*.nw,kw,tw.
2. “Music Therapy”/ or Music/
3. (“use*” or usage* or function* or intention* or intent? or utiliz*).tw, kw.
4. exp Intention/
5. (Psychometric* or (psycholog* adj3 (metric* or test* or measure* or tool* or instrument* or scale* or questionnaire*))).tw, kw.
6. Psychometrics/
7. (1 or 2) and (3 or 4) and (5 or 6)
8. limit 7 to yr =”2000-2023”

*n* = 209

20230405

RILM (Ebsco)

1. TI (“use*” OR usage* OR function* OR intention* OR intent? OR utiliz* OR engage* OR affect* OR respond* OR respons*) OR AB (“use*” OR usage* OR function* OR intention* OR intent? OR utiliz* OR engage* OR affect* OR respond* OR respons*)
2. TI (Psychometric* OR (psycholog* N3 (metric* or test* or measure* or tool* OR instrument* OR scale* OR questionnaire*))) OR AB (Psychometric* OR (psycholog* N3 (metric* or test* or measure* or tool* OR instrument* OR scale* OR questionnaire*)))
3. S1 AND S2
4. limit 3 to yr =”2000-2023”
5. Limit to Academic Journals
6. Limited to English only

*n* = 143

20230405

CINAHL Ultimate (Ebsco)

1. TI (music*) OR AB (music*)
2. (MH “Music”) OR (MH “Music Therapy”)
3. TI (“use*” OR usage* OR function* OR intention* OR intent? OR utiliz* OR engage* OR affect* OR respond* OR respons*) OR AB (“use*” OR usage* OR function* OR intention* OR intent? OR utiliz* OR engage* OR affect* OR respond* OR respons*)
4. TI (Psychometric* OR (psycholog* N3 (metric* or test* or measure* or tool* OR instrument* OR scale* OR questionnaire*)))
5. (MH “Psychometrics”)
6. (S1 OR S2) AND S3 AND (S4 OR S5)
7. Limit to yr =”2000-2023”
8. Limit to Academic Journals
9. Limited to English only

*n* = 39

20230405

Music Index Online (Ebsco)

1. TI (“use*” OR usage* OR function* OR intention* OR intent? OR utiliz* OR engage* OR affect* OR respond* OR respons*) OR AB (“use*” OR usage* OR function* OR intention* OR intent? OR utiliz* OR engage* OR affect* OR respond* OR respons*)
2. TI (Psychometric* OR (psycholog* N3 (metric* or test* or measure* or tool* OR instrument* OR scale* OR questionnaire*))) OR AB (Psychometric* OR (psycholog* N3 (metric* or test* or measure* or tool* OR instrument* OR scale* OR questionnaire*)))
3. (ZU “psychometrics”)
4. S1 AND (S2 OR S3)
5. Limit 4 to yr =”2000-2023”
6. Limit to Academic Journals
7. Limited to English only

*n* = 246

20230405

Music Periodicals Database (Proquest)

1. title(((“use*” OR usage* OR function* OR intention* OR intent? OR utiliz* OR engage* OR affect* OR respond* OR respons*))) OR abstract(((“use*” OR usage* OR function* OR intention* OR intent? OR utiliz* OR engage* OR affect* OR respond* OR respons*)))
2. TI (Psychometric* OR (psycholog* NEAR/3 (metric* or test* or measure* or tool* OR instrument* OR scale* OR questionnaire*))) OR AB (Psychometric* OR (psycholog* NEAR/3 (metric* or test* or measure* or tool* OR instrument* OR scale* OR questionnaire*)))
3. 1 AND 2
4. limit 3 to yr =”2000-2023”
5. Limit to Academic Journals
6. Limited to English only

*n* = 74

20230405

PsyArXiv

(psychometric OR (psycholog* AND (metric* or test* or measure* or tool* OR instrument* OR scale* OR questionnaire*))) AND music*

## Appendix 2

**Table:** Overview of all Categories and Inventories.

Functions of music	Music engagement	Clinical assessment	Music-based intervention	Musicians’ health	Music processing	Perception of self and others
Adaptive Functions of Music Listening Scale (AFML; Groarke & Hogan, 2020)	Exposure to Music in Childhood Inventory	Dementia Coding System (DeCS; Hillebrand et al., 2023)	Assessment of Parenting Competencies, revised (APC-R; Jacobsen & McKinney, 2015)	Evaluation of the Ability to Sing Easily (EASE; Phyland et al., 2013)	Absorption in Music Scale (AIMS; Sandstrom & Russo, 2011)	Caregiver Confidence using Music Scale (CCuMS; Kim et al., 2021)
Barcelona Music Reward Questionnaire (BMRQ; Mas-Herrero et al., 2013)	Goldsmith’s Musical Sophistication Index (Gold-MSI; Mullensiefen et al., 2014)	Music Attentiveness Screening Assessment, Revised (MASA-R; Waldon et al., 2016)	Interpersonal Music-Communication Competence Scale (Hald et al., 2017)	Music Performance Anxiety Inventory for Adolescents (MPAI-A; Osborne & Kenny, 2005)	Music- and Image-related Typicality Scales (Cohrdes & Kopiez, 2015)	Children and Adolescent Chorister Engagement Survey (CACES; Zhukov et al., 2021)
Brief Music in Mood Regulation Scale (B-MMR; Saarikallio, 2012)	Music@Home Questionnaire (Politimou et al., 2018)	Music-based Autism Diagnostics (MUSAD; Bergmann et al., 2015)	Music Assessment Tool for Mechanically Ventilated Patients (MAT; Chlan & Heiderscheit, 2009)	Musician Occupational Stress Scale (MOSS; King et al., 2019)	Music-empathizing-systemizing (Kreutz et al., 2008)	Music Self-perception Inventory (MUSPI; short form; Morin et al., 2016)
Brunel Music Rating Inventory-2 (Karageorghis et al., 2006)	Music Engagement Questionnaire (MusEQ; Vanstone et al., 2016)	Music-based Attention Assessment (MAA; Jeong & Lesiuk, 2011)	Music Therapy Engagement Scale for Dementia (MTED; Tan et al., 2019)	Musician’s arousal regulation imagery scale (MARIS; Finch et al., 2021)	Music Receptivity Scale (MRS; George & Ilavarasu, 2021)	Musical Humility Scale (Coppola et al., 2021)
Emotion Regulation Strategies for Artistic Creative Activities Scale (ERS-ACA; Fancourt et al., 2019)	Music Experience Questionnaire (MEQ; Werner et al., 2006)	Music-based Attention Assessment-Revised (MAA-R; Jeong, 2013)	Music Therapy Self-Rating Scale (MTSRS; Meadows et al., 2015)	Musculoskeletal Pain Intensity and Interference Questionnaire (Berque et al., 2014)		Musical Self-concept Inquiry (MUSCI; Fiedler & Spychiger, 2017)
Eudaimonic Functions of Music Listening Scale (Groarke & Hogan, 2020)	Music in Everyday Life (MEL; Gottfried et al., 2018)	Music Cognitive Test (MCT; Mangiacotti et al., 2023)	The Music Therapy Session Assessment Scale (MT-SAS; Raglio et al., 2017)	Psychosocial risks questionnaire for musicians (PRQM; Jacukowicz & Wezyk, 2018)		
Healthy-Unhealthy Music Scale (HUMS; Saarikallio et al., 2015)	Music Use and Background Questionnaire (MUSEBAQ; Chin et al., 2018)	Music in Dementia Assessment Scales (MiDAS; McDermott et al., 2015)	Pediatric Inpatients Music Therapy Assessment Form (PIMTAF; Douglass, 2006)	The Technical Ability and Performing Scale (TAPS; Ramella et al., 2022)		
Hedonic and Eudaimonic Motivations for Music Scale (HEMM; Powell et al., 2023)		Music Therapy Assessment Tool for Advanced Huntington’s Disease (MATA-HD; O’Kelly & Bodak, 2016)	Psychosocial Risks Questionnaire for Musicians (PRQM; Jacukowicz & Wezyk, 2018)			
Individual and Community Empowerment Inventory (ICE; Travis & Bowman, 2015)		Playing-in-Touch Questionnaire (PiT; Politi et al., 2012)	The Technical Ability and Performing Scale (TAPS; Ramella et al., 2022)			
Motives for Listening to Music Questionnaire (MLMQ; Kuntsche et al., 2016)						
Music in Mood Regulation (MMR; Saarikallio, 2008)						
The Music Use Questionnaire (MUSE; Chin & Rickard, 2012)						
Music Mood-Regulation Scale (MMRS; Hewston et al., 2009)						
Musical Activity and Well-being Scale (Krause et al., 2018)						
Musical Engagement (Hollebeek et al., 2016)						
Rating of Experienced Social Personal and Cultural Themes of Music Functions (RESPECT-Music; Boer et al., 2012)						
